# Hyperprogression to camrelizumab in a patient with esophageal squamous cell carcinoma harboring EGFR kinase domain duplication

**DOI:** 10.1136/jitc-2020-000793

**Published:** 2020-06-23

**Authors:** Wei Wang, Meihong Wu, Minglu Liu, Zhengqing Yan, Guoqiang Wang, Dongliang Mao, Mei Wang

**Affiliations:** 1 Department of Oncology, Changhai Hospital of Shanghai, Shanghai, China; 2 Department of Radiology, Changhai Hospital of Shanghai, Shanghai, China; 3 The Medical Department, 3D Medicines Inc, Shanghai, China; 4 Department of Oncology, North Ruijin Hospital, Shanghai Jiao Tong University, Shanghai, China

**Keywords:** immunotherapy, genetic markers, tumor biomarkers, antibodies, neoplasm

## Abstract

**Background:**

Previous studies have reported that the amplification of some genes, such as *Murine Double Minute 2 or 4* and *Epidermal Growth Factor Receptor* (*EGFR*), may be related to hyperprogressive disease (HPD). Exploring somatic gene alterations might be an effective method to predict HPD. Herein we characterize the somatic alterations in a patient with esophageal squamous cell carcinoma (ESCC) who developed HPD to investigate the potential origins of HPD.

**Case presentation:**

A man in his mid-40s was diagnosed with ESCC. After the failure of first-line treatment with cisplatin and docetaxel, the patient participated in a phase III randomized, open, multicenter clinical trial (CTR20170307) and subsequently received camrelizumab. After 4 weeks of immunotherapy, the tumor size increased by 79% compared with baseline imaging; the progressive pace was 2.5-fold higher than preimmunotherapy, and a new liver metastasis appeared. A rare EGFR exon 2–28 duplication was discovered in both preimmunotherapy and postimmunotherapy tumor tissues.

**Conclusion:**

This is the first report on a patient with ESCC harboring rare *EGFR* kinase domain duplication in exons 2–28 and developing HPD in the process of camrelizumab treatment. This case suggested that *EGFR* kinase domain duplication might be associated with HPD. Administration of immune checkpoint inhibitor monotherapy in this subgroup of patients harboring *EGFR* kinase domain duplication should be performed with caution. These results need to be further confirmed in a larger cohort of patients.

## Introduction

Immune checkpoint inhibitors (ICIs), including programmed cell death 1 (PD-1), programmed cell death ligand 1 (PD-L1) and cytotoxic T-lymphocyte-associated antigen (CTL)-4, have a positive effect on cancer treatment via reconstructing efficient antitumor T-cell response. Compared with traditional chemotherapy, ICIs, as a single agent or in combination, can bring a clear overall survival (OS) benefit, produce durable responses and have good tolerability in patients. Until now, a few ICIs, such as pembrolizumab and nivolumab, have been approved by the Food and Drug Administration in melanoma, non-small-cell lung cancer (NSCLC), colorectal cancer, gastric cancer and esophageal carcinoma (EC), and more drugs are awaiting to be approved. From this perspective, the future of ICI therapy seems to be bright.^1 2^ However, some recent studies have shown that ICIs do more harm than good since tumor growth acceleration occurs in a subset of patients, known as hyperprogressive disease (HPD).^3–5^ According to the work of Kato *et al*, HPD was defined as disease progression by RECIST V.1.1 criteria with a ≥2 fold increase in tumor growth rate compared with preimmunotherapy treatment, >50% increase in tumor load and time-to-treatment failure of <2 months during immunotherapy.^6^ A few studies suggested that the appearance of HPD was strongly associated with shorter OS and progression-free survival (PFS).^7–9^ Such HPD phenomenon was observed across many advanced cancers types, such as head and neck squamous cell carcinoma,^10^ NSCLC,^7^ urothelial carcinoma^11^ and gastrointestinal tract cancer.^12 13^ Considering the deleterious effects of HPD, we find it is important to find out patients who may develop HPD before ICI treatments.

Previous studies suggested that HPD was associated with many factors, such as advanced age,^14 15^ the number of metastatic sites^15^ or lactate dehydrogenase levels.^15^ However, the correlation between HPD and the aforementioned factors was still controversial.^7 9^ Additionally, several genomic alterations had been found to be correlated with HPD, such as murine double minute 2 or 4 (*MDM2*/*MDM4*) amplification,^6^ epidermal growth factor receptor (*EGFR*) amplification, the amplification of several genes on chromosomes 11q13-*CCND1, FGF19, FGF3, and FGF4*.^16^ Moreover, a few cases indicated that *EGFR*-mutated tumors (*EGFR* E746-A750 del and T790M mutation^6^ or *EGFR* exon 20 insertion mutation and *MYC* amplification^17^) also had a less satisfactory rate of response to ICIs and developed rapid progression. To sum up, the characterization of somatic gene alterations might be an effective method to predict HPD. Therefore, we characterized the somatic alterations in a patient with esophageal squamous cell carcinoma (ESCC) who developed HPD to investigate the potential origins of HPD.

## Case presentation

A man in his mid-40s was diagnosed as ESCC. The patient underwent esophagectomy via thoracoabdominal approach without chemoradiotherapy, and his tumor node metastasis was pT1bN2M0. One year later, metastatic lesions were observed in the mediastina, the left axilla and the abdominal cavity. Then, he received first-line treatment with six cycles of cisplatin (60 mg, day 1; 40 mg days 2 and 3) plus docetaxel (140 mg, day 1).

**Figure 1:**
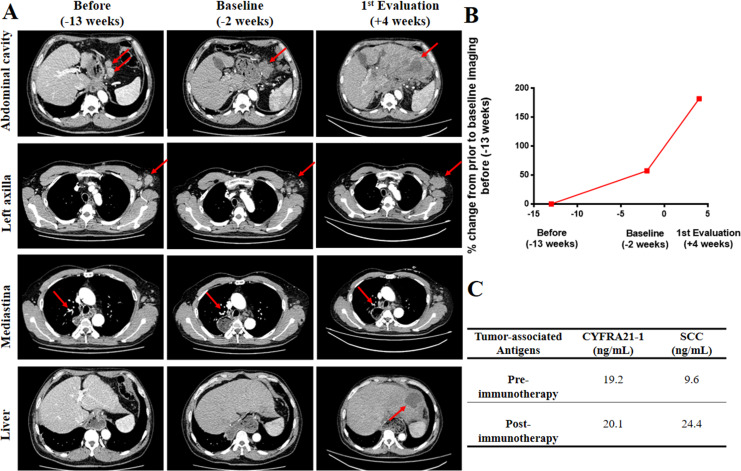
Case study of a patient in his mid-40s with HPD during immunotherapy. (A) CT scans were performed 13 weeks before starting anti-PD-1 treatment (column 1), at baseline (2 weeks before starting immunotherapy, column 2), and at first evaluation (4 weeks after starting immunotherapy, column 3). CT scans from lines 1 to 4 revealed the changes in lymph nodes in the abdominal cavity, left axilla and mediastina, respectively. New liver lesion appeared. The red arrows indicate tumer lesions.(B) Rate of change in growth pattern in the patient, who developed HPD to camrelizumab. Compared with the tumor image (−13 weeks), the tumor lesions at baseline (−2 weeks) and at first evaluation (4 weeks after starting immunotherapy) showed approximately 57% and 181% increases (79% increase compared with baseline imaging), respectively; 2.5-fold increase in progressive pace compared with preimmunotherapy. (C) Changes in tumor-associated antigens before and after immunotherapy. CYFRA21-1, cytokeratin-19 fragment; HPD, hyperprogressive disease; PD-1, programmed cell death 1; SCC, squamous cell carcinoma.

After progression, the patient participated in a phase III randomized, open, multicenter study comparing camrelizumab (PD-1 blockade) to chemotherapy of physician's choice for patients with advanced EC (CTR20170307). During the clinical trial, he was assigned to receive camrelizumab (400 mg d1). After 4 weeks, the CT scans demonstrated a new liver metastasis and enlarged lymph nodes in the left axilla and abdominal cavity compared with baseline imaging (figure 1A). The tumor size increased by 79% compared with baseline imaging; the progressive pace was 2.5-fold higher than preimmunotherapy. (figure 1B). The progressive disease was evaluated as HPD according to the criteria defined by Kato and colleagues.^6^ The pathological analysis of new liver metastasis indicated ESCC. Additionally, squamous cell carcinoma (SCC) antigens, one of the tumor-associated antigens before and after immunotherapy were 9.6 and 24.4 ng/mL respectively (figure 1C). After the failure of anti-PD-1 therapy, three cycles of gemcitabine (1.8 g, days 1 and 5) and nedaplatin (70 mg, days 1 and 2) were administrated and then stopped because of pain. Subsequently, best supportive care was given afterward, yet the patient died of rapid systematic progression.

**Figure 2:**
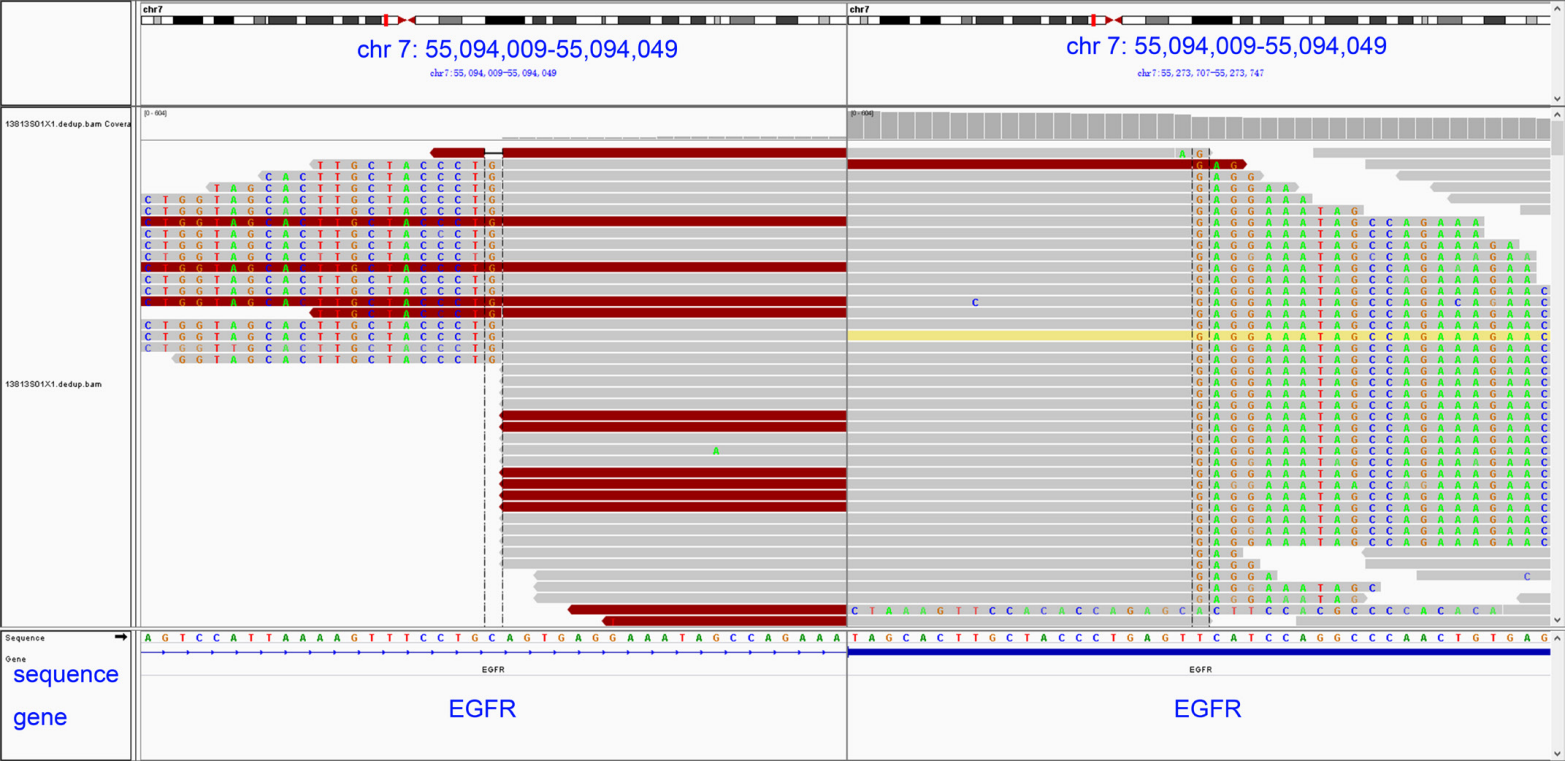
Visualization of atypical *EGFR-KDD* events occurring in exons 2–28 using the Integrative Genomics Viewer browser. EGFR, epidermal growth factor receptor.

In order to investigate the mechanism of HPD, preimmunotherapy and postimmunotherapy tissues were subjected to next-generation sequencing in a College of American Pathologists-certified and Clinical Laboratory Improvement Amendment-accredited laboratory, respectively.^18^ Before immunotherapy, the tumor mutation burden (TMB) of the tumor tissue was 3.23, and PD-L1 expression was observed in less than 1% of tumor cells (PD-L1 negative). The somatic alteration EGFR exon 2–28 duplication existed in both preimmunotherapy and postimmunotherapy tumor tissues (figure 2 and table 1), which suggested that it might be associated with HPD.

**Table 1:** Somatic alterations before and after immunotherapy.

	Preimmunotherapy	Postimmunotherapy
Somatic alterations	*EGFR* exon2-28 dup 3545 bp *EGFR* amplifications *CCND1* amplifications *FGF19* amplifications *FGF3* amplifications *FGF4* amplifications *MCL1* amplifications *TP 53* Exon5 p.C135Afs*35	*EGFR* exon2-28 dup 3545 bp *TP53* Exon5 p.C135Afs*35 *RB1* reduced copy number

EGFR, epidermal growth factor receptor.

## Discussion

In this case, a man in his mid-40s was diagnosed as ESCC. After the failure of first-line chemotherapy, he participated in a phase III clinical trial and was assigned to receive camrelizumab. After 4 weeks of immunotherapy, the tumor size increased by 79% compared with baseline imaging; the progressive pace was 2.5-fold higher than preimmunotherapy; and a new liver metastasis appeared. The somatic alteration EGFR exon 2–28 duplication (existed in both preimmunotherapy and postimmunotherapy tumor tissues) was thought to be associated with HPD, which was never reported before. Reported HPD prediction factors include advanced age and more than two metastatic sites.^7 14^ However, sufficient evidence is not available. The current case had more than two metastases, but he was under 65 years old.

Considering that patients who developed HPD usually had worse OS and PFS compared with patients without HPD, an increasing attention has been paid on the relationship between somatic gene alterations and HPD during immunotherapy. *MDM2*/*MDM4* is an important negative regulator of the tumor suppressor p53 by inhibiting its transcriptional activity and degrading it via ubiquitination. The *MDM*2/*MDM*4 amplification is significantly correlated with HPD. Kato *et al* provided a hypothesis that interferon (IFN)-γ elevated by ICIs in turn activates JAK-STAT signaling and interferon regulatory factor-8 expression, which can bind the promoter of gene *MDM2* and result in the sequent hyperexpression of *MDM2* in the patients harboring *MDM2* amplification.^6^ Singavi and coworkers reported that one patient with ESCC harboring *MDM*4 amplification developed HPD during immunotherapy treatment.^16^ Besides, the amplification of EGFR and genes located on chromosome 11q13 (*CCND1*, *FGF19*, *FGF3*, and *FGF4*) might be also associated with HPD.^16^ In the present case, some gene amplifications, including *CCND1*, *FGF19*, *FGF3*, *FGF4* and *EGFR*, existed in preimmunotherapy tissue, but such genes amplications disappeared in postimmunotherapy tissue, which suggests such genes may not be associated with the HPD of this patient.

Previous works demonstrated that the inactivated mutations of *RB1* and *TP53* usually occurred in the *EGFR*-mutant lung adenocarcinomas that transformed to small-cell lung cancer and other neuroendocrine carcinomas.^19^ Recently, TP53 mutation is associated with the significant clinical benefit to ICIs in NSCLC by cell signal pathways, such as cell cycle, DNA replication and damage repair.^20^Additionally, the comutation *RB1* and *TP53* existed in tumor immune microenvironment type I (high PD-L1/high CD8A) bladder tumors, which could significantly activate T-effector and IFN-γ signature.^21^ In view of the results mentioned earlier, the alterations of TP53 and RB1, which are also found in postimmunotherapy tissues, may not be suggested to contribute to ICI-related HPD.

In the case, the somatic alteration EGFR exon 2–28 duplication, subtype of EGFR-Kinase Domain Duplication*(*KDD), was associated with HPD. EGFR-KDD was first reported by Gallant *et al* in 2015.^22^ Such EGFR-KDDs are often observed in lung, brain and soft tissue cancers. The canonical EGFR-KDD is an in-frame tandem duplication of EGFR exons 18–25 (11/13). Besides, the unusual events, such as *EGFR* exons 17–25 duplication (1/13) and *EGFR* exons 14–26 duplication (1/13), are also reported.^23^ The *EGFR* exon 2–28 duplication is first reported in this case. Although EGFR-KDD has been known as one of the oncogenic drivers which can activate EGFR signaling via forming an intramolecular dimer, it has never been found in EC. Except *EGFR-KDD*, some tumors harboring other *EGFR* alterations, such as *EGFR* exon 20 insertion mutation, *EGFR* E746-A750 del and *EGFR* T790M mutation, also developed HPD during immunotherapy. A few studies are trying to explain the relationship between EGFR mutation and HPD. For example, previous study demonstrated that the EGFR activation could upregulate the expression of PD-1, PD-L1 and CTLA-1, promoting immune escape. In another study, it was found that anti-PD-1 agents could boost EGFR-mutant tumor growth through interaction with M2-like macrophages.^24^ This case reminded that administration of ICI monotherapy in this subgroup of patients harboring *EGFR-KDD* should be performed with caution in future clinical practice. Predictive biomarkers of response to immunotherapy, including positive factors (PD-L1 and TMB) and negative factors (EGFR and *MDM2/MDM4* 25), are needed before receiving immunotherapy.^25^ Ferrara *et al* suggested that addition of chemotherapy to ICIs was a potential method to overcome the PD-1/PD-L1 inhibitor resistance and ICI-related HPD.^7^ Such results need to be confirmed in further investigations.

## Conclusion

In summary, the present case is the first report describing a patient with ESCC harboring EGFR-KDD who developed HPD during ICI treatment. In this case, EGFR exon 2–28 duplication was thought to be associated with HPD. Administration of ICI monotherapy should be performed with caution in this subgroup of patients harboring EGFR-KDD. The results should be confirmed in a larger cohort of patients, and the potential mechanism by which EGFR-KDD caused HPD during immunotherapy should also be investigated. Further analysis of such cases that developed HPD during ICI therapy might be helpful to find out putative predictive biomarkers for HPD.

### Methods

The preimmunotherapy and postimmunotherapy tissue DNA alterations and TMB in a patient with ESCC who developed HPD were characterized via NGS 387 or 417 gene panel (3DMed, Shanghai, China).^18^ The PD-L1 expression was measured via SP142 and 22C3 immunohistochemistry assays, respectively.
